# The In Vitro α-Glucosidase Inhibition Activity of Various Solvent Fractions of *Tamarix dioica* and ^1^H-NMR Based Metabolite Identification and Molecular Docking Analysis

**DOI:** 10.3390/plants10061128

**Published:** 2021-06-02

**Authors:** Aamir Niaz, Ahmad Adnan, Rashida Bashir, Muhammad Waseem Mumtaz, Syed Ali Raza, Umer Rashid, Chin Ping Tan, Tai Boon Tan

**Affiliations:** 1 Department of Chemistry, GC University Lahore, Lahore 54000, Pakistan; aamirzar91@gmail.com (A.N.); chemstone@yahoo.com (S.A.R.); 2 Department of Chemistry, University of Education Lahore, Lahore 54000, Pakistan; rashida.bashir@ue.edu.pk; 3 Department of Chemistry, University of Gujrat, Gujrat 50700, Pakistan; muhammad.waseem@uog.edu.pk; 4 Institute of Advanced Technology, Universiti Putra Malaysia, Serdang 43400, Selangor, Malaysia; 5 Department of Food Technology, Faculty of Food Science and Technology, Universiti Putra Malaysia, Serdang 43400, Selangor, Malaysia; tancp@upm.edu.my; 6 Department of Food Services and Management, Faculty of Food Science and Technology, Universiti Putra Malaysia, Serdang 43400, Selangor, Malaysia; taiboon_tan@upm.edu.my

**Keywords:** antioxidant, α-glucosidase, *Tamarix dioica*, ^1^H-NMR, metabolites, docking

## Abstract

The *Tamarix dioica* (*T. dioica*) is widely used medicinal plant to cure many chronic ailments. *T. dioica* is being used to manage diabetes mellitus in traditional medicinal system; however, very little scientific evidence is available on this plant in this context. The current study involves the fractionation of crude methanolic extract of *T. dioica* using *n*-hexane, ethyl acetate, chloroform, and *n*-butanol. The screening for antioxidant activity using 2,2-diphenyl-1-picrylhydrazyl (DPPH) assay was carried out. The in vitro antidiabetic potential was assessed by measuring α-glucosidase inhibition. Total phenolic and flavonoid contents were also determined for each fraction. The metabolites were identified using highly sensitive and emerging ^1^H-NMR technique. The results revealed the ethyl acetate fraction as the most potent with DPPH scavenging activity of 84.44 ± 0.21% and α-glucosidase inhibition with IC_50_ value of 122.81 ± 2.05 µg/mL. The total phenolic and flavonoid content values of 205.45 ± 1.36 mg gallic acid equivalent per gram dried extract and 156.85 ± 1.33 mg quercetin equivalent per gram dried extract were obtained for ethyl acetate fraction. The bucketing of ^1^H-NMR spectra identified 22 metabolites including some pharmacologically important like tamarixetin, tamaridone, quercetin, rutin, apigenin, catechin, kaempferol, myricetin and isorhamnetin. Leucine, lysine, glutamic acid, aspartic acid, serine, and tyrosine were the major amino acids identified in ethyl acetate fraction. The molecular docking analysis provided significant information on the binding affinity among secondary metabolites and α-glucosidase. These metabolites were most probably responsible for the antioxidant activity and α-glucosidase inhibitory potential of ethyl acetate fraction. The study ascertained the ethnomedicinal use of *T. dioica* to manage diabetes mellitus and may be a helpful lead towards naturopathic mode for anti-hyperglycemia.

## 1. Introduction

Many plants from genus *Tamarix* are frequently used to treat many health disorders in conventional mode of treatment. The genus *Tamarix* of family Tamaricaceae has almost 60 species and the majority of them are flowering plants. The genus *Tamarix* is known to feed more than 250 species of animals including domestic cattle. The *Tamarix* spp. is traditionally used to treat diabetes, dental problems and gastrointestinal disorders by the local inhabitants in Asian and African countries [1]. The *Tamarix dioica* (*T. dioica*) is an important species of genus *Tamarix. T. dioica* is evergreen small tree ranging from 1 to 18 m in height. The flowers are purple in color and, in Pakistan, this plant is widely distributed in Khyber Pakhtunkhwa and Sindh provinces [2].

As *T. dicoca* of family Tamaricaceae is native to many areas of Pakistan and is known as Ghaz or Khagal. It is an ever-green shrub like tree and is known to possess many medicinal properties including antimicrobial, diuretic, carminative and hepatoprotective. A study on qualitative phytochemical analysis of *T. dioica* revealed the presence of steroids, phenols, tannins, flavonoids and saponins where alkaloids and amino acids were absent [3,4]. Another study reported the HPLC-DAD analysis of crude hydrometahnolic extract of *T. dioica* and proved the presence of gallic acid, rutin, myricetin and apigenin [5]. *T. dioica* is used to cure diabetes in traditional medicinal system but scientifically confirmed evidence is lacking in this regard.

Diabetes mellitus (DM) is fast spreading endocrine metabolic dysfunction or disease associated with persistently higher blood glucose level. DM is expected to target 5.4% of world’s population by 2025 [6]. The disturbed metabolic activity and resultant oxidative stress due to DM propagation are responsible for several side complications like cardiovascular disorders, retinal damage, renal and vascular dysfunctions [7]. Many synthetic drugs are available to reduce the blood glucose level, but these compounds have some serious side effects like hypoglycemia, insulinoma, gastrointestinal problems, kidney dysfunction and heart problems [8]. Moreover, drug resistance, loss of efficacy and high cost of synthetic drugs are important factors reflecting the need for alternative medicine with low or no toxicity [9]. 

Reactive oxygen species (ROS) are produced in the body during metabolic activity. The antioxidant defense system of body balances the ROS under normal metabolic conditions. However, under certain circumstances or diseased conditions, the ROS level may increase to considerable extent for the development of the state of oxidative stress. The oxidative stress plays an important role in DM complications which make health outcomes more adverse. ROS mediated important pathways like polyol pathway flux, glycated end products, activation of receptor sites for glycated end products and oxidative damage to beta cells of pancreas are the leading cause of DM propagated complications [10,11]. 

The side effects of antidiabetic drugs urge to develop novel strategies for DM cure and management. Alternatively, the α-glucosidase inhibition may an encouraging aspect to reduce the postprandial blood glucose level. The α-glucosidase is the enzyme which hydrolyzes carbohydrates to glucose in brush borderlines of intestine for consequent absorption [12]. Medicinal plants are being used to cure many ailments since long. Plants contain phytochemicals or metabolites which have numerous medicinal properties. These compounds are mainly phenolics and flavonoids in nature and have ability to scavenge free radicals or ROS and can inhibit α-glucosidase enzyme to control blood glucose level. The plants can provide a dual charge in terms of antioxidant potential and dietary enzyme inhibitory action. Many plants were investigated for their antioxidant and antidiabetic activities and studies correlated the antioxidant activity of plants to blood glucose lowering activity [13,14]. 

The antidiabetic activities of plant extracts were most probably due to polyphenols present in plant extracts as reported by previous investigations and inhibition of α-glucosidase was assigned as the main target for management of high postprandial blood glucose level [15,16,17]. The phyto-constituents or secondary metabolites of plants are one of the highly imperative fields to explore the novel compounds or groups of substances to cure diseases. The identification of metabolites is very important to predict the medicinal potential of a particular plant. Various techniques are in the field; however, recent advances in ^1^H-NMR based metabolite profiling is serving as an excellent tool to explore the compounds on the basis of their chemical shift values [18,19].

The identified metabolites of plants can be evaluated for possible structural modification in enzymes to inhibit or reduce their activities. Recent advances in computation chemistry and biology have gained enormous importance and molecular docking is one of the leading tools in this regard. Studies have confirmed the role of docking analysis to predict the potential amino acid residues, which may be of great therapeutic importance [20,21]. The secondary metabolites of plants may be docked in various enzymes to evaluate their binding affinity which serves as supporting evidence for in vitro findings like percentage inhibition or IC_50_ values [14,22]. 

The *T. dioica* is potentially used in local medicinal system to treat DM but no well-established experimentally verified evidence is available in this context. It was necessary to explore the *T. dioica* for possible or latent antidiabetic properties. The current work was designed to evident the free radical scavenging and α-glucosidase inhibitory potential of various fractions of *T. dioica* in vitro along with ^1^H-NMR based metabolite profiling to identify the possible pharmacologically active metabolites. The ability of metabolites to interact with amino acid residues of α-glucosidase was computed using molecular docking.

## 2. Results

### 2.1. Total Phenolic and Flavonoid Contents

The results of total phenolic contents (TPC) and total flavonoid contents (TFC) are given in Table 1. The results indicated that highest TPC and TFC were observed for ethyl acetate fraction followed by chloroform. The one-way ANOVA based statistical comparison showed that the TPC and TFC values for ethyl acetate fraction were higher than remaining fractions to a significant level (*p* < 0.05). The second highest TPC and TFC were observed for chloroform fraction and lowest yields of TPC and TFC were obtained with *n*-hexane.

### 2.2. Antioxidant Activity

**Figure 1:**
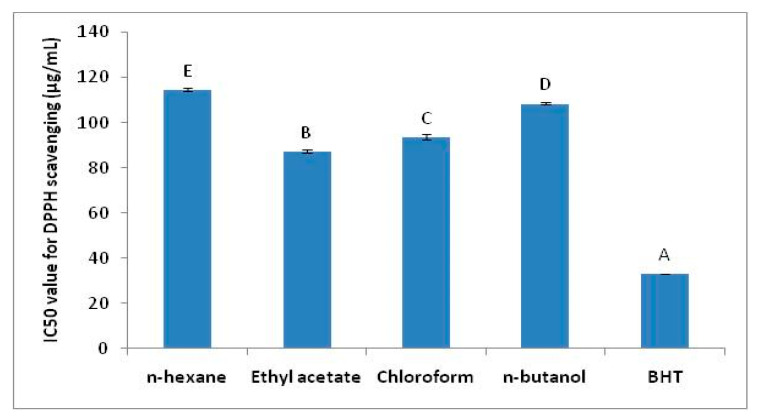
IC_50_ values of DPPH radical scavenging for different fractions of *T. dioica*. The ethyl acetate fraction showed lowest IC_50_ and significantly higher among all fractions. The level of significance difference was represented by alphabets. Values having different alphabets (A–E) were statistically significant (*p* < 0.05).

The results of DPPH radical scavenging are given in Figure 1. The IC_50_ values of DPPH radical scavenging for various extract fractions of *T. dioica* clearly demonstrated that ethyl acetate fraction was the most efficient one to scavenge DPPH free radical with IC_50_ of 87.23 ± 0.70 µg/mL among all. This value was significantly higher among all fractions as indicated by *p* < 0.05. This value of DPPH scavenging was considerable but could not reach the value exhibited by BHT used as pure standard antioxidant. Chloroform fraction also showed reasonable antioxidant activity but significantly less effective as compared to ethyl acetate fraction. The lowest DPPH activity was exhibited by *n*-hexane fraction with IC_50_ 114.55 ± 0.76 µg/mL.

### 2.3. The α-Glucosidase Inhibition Assay

**Figure 2:**
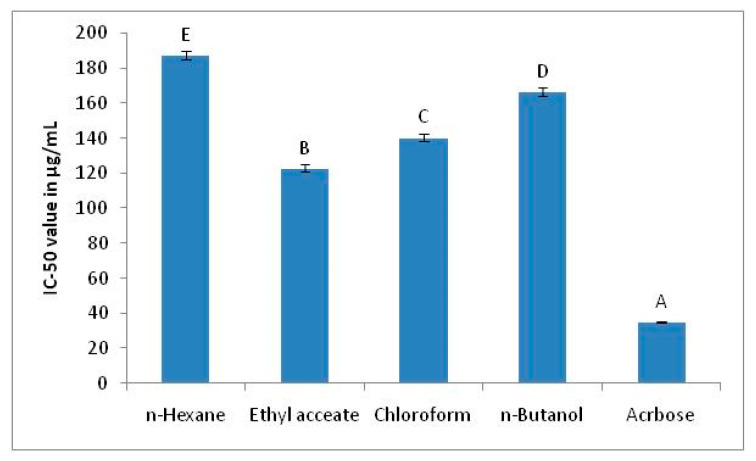
IC_50_ values for α-glucosidase inhibition by different solvent fractions of *T. dioica*. The ethyl acetate fraction showed lowest IC_50_ and significantly higher among all fractions. The level of significance difference was represented by alphabets. Values having different alphabets (A–E) were statistically significant (*p* < 0.05).

The inhibition of α-glucosidase was checked by the plant extract fractions to assess the in vitro antidiabetic activity. The findings of α-glucosidase inhibition in terms of IC_50_ values are given as Figure 2. 

The findings of α-glucosidase inhibition by extract fractions of *T. dioica* are given in Figure 2. The results demonstrated that ethyl acetate fraction showed maximum inhibition of α-glucosidase with IC_50_ value of 122.81 ± 2.05 µg/mL. The second highest enzyme inhibition was shown by chloroform fraction. The statistical analysis confirmed that ethyl acetate fraction exhibited significantly higher α-glucosidase inhibition among all extracts (*p* < 0.05). The n-hexane fraction was the least responsive towards enzyme inhibition when compared to another fraction.

### 2.4. Metabolite Profiling

**Figure 3:**
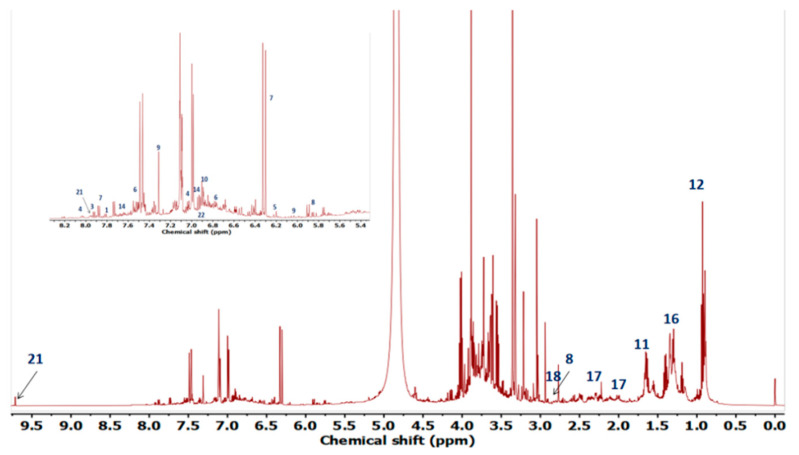
^1^H-NMR spectra of *Tamarix dioica* ethyl acetate extract indicating the chemical shift values for compound identification.

**Figure 4:**
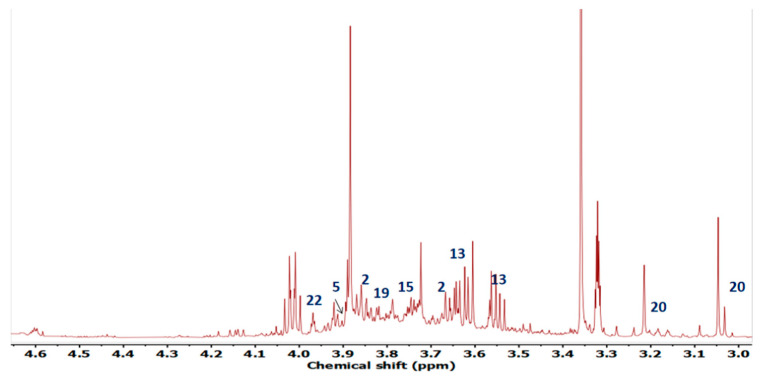
Annotated compounds in the carbohydrate region of the ^1^H-NMR spectra.

The ^1^HNMR spectrum of extract is given as Figure 3. The annotation of signals corresponding to metabolites is given as Figure 4.

The compounds were identified on the basis of their chemical shift values and *J* resolved values for duplets and multiplets. The identification revealed that there were compounds of diverse chemical classes including flavonoids, organic acids, and amino acids.

The 22 metabolites were identified on the basis of spectral information. The detail of identified compounds and their characteristic peak data is given in Table 2.

### 2.5. Molecular Docking Data

**Figure 5:**
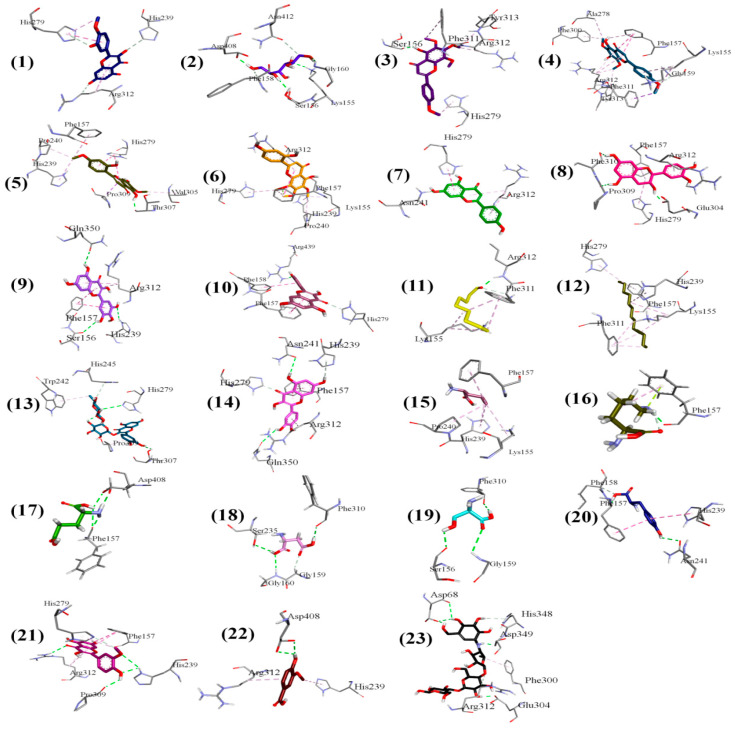
The 3D interaction plots of identified phytochemicals of ethyl acetate fraction from *Tamarix dioica* and acarbose with α-glucosidase.

**Figure 6:**
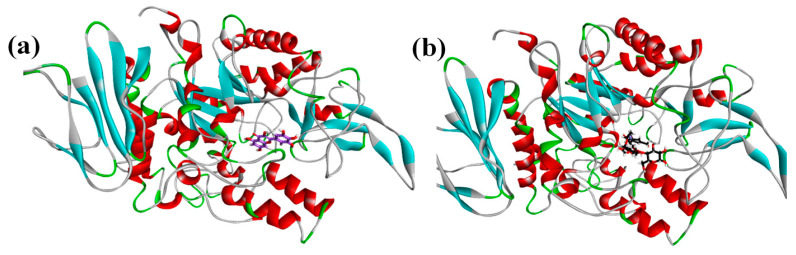
The Ribbon Diagram of Myricetin (**a**) and Acarbose (**b**) in active pockets of α-glucosidase.

The phytochemicals identified by NMR were docked with the homology modelled 3D structure of alpha-glucosidase. The binding energies of protein-phytochemical interactions were calculated by MOE software. The 3D Interaction plots of phytochemicals and acarbose with α-glucosidase are given as Figure 5 and ribbon diagram of myricetin and acarbose in active site pockets of α-glucosidase are given as Figure 6.

The binding energies of all the identified phytochemicals along with their interactions with the active site residues of the α-glucosidase are presented in Table 3. Acarbose (standard anti-diabetic drug) was used as a reference to estimate the relative effectiveness of identified phytochemicals as α-glucosidase inhibitors. Acarbose exhibited binding energy of −16.4212 kJ/mol with alpha-glucosidase, while binding energies of the phytochemicals were in the range of −8.0170 to −15.3993 kJ/mol. The lowest binding energy was presented by Myricetin (−15.399 kJ/mol); however, binding energies of Quercetin and Rutin were also comparable to the binding energy of acarbose. 

The conventional H-bonding was observed among metabolites and amino acid residues of enzyme as shown in Table 3. Some other interactions like pi-pi stacking, pi-sigma and C-H bonding were also noticed. The ARG312 and SER156 sites appeared as interaction points for multiple compounds and, therefore, might be of interest regarding drug development. 

## 3. Discussion

The plant-based medicinal system is widely adopted to treat many ailments and most of the therapeutic effects of plants and their extracts depend upon the polyphenolic system. The concentration of phenolic depends upon the extraction conditions but the role of solvent system used for extraction is imperative. The variations observed for TPC and TFC in the current study were most probably due to variation in the solvent system used for extraction. The polarity of ethyl acetate seemed most compatible with the nature of compounds present in crude methanolic extract of *T. dioica* [23]. TPC and TFC are considered as principal components responsible for various medicinal activities shown by plants. The higher amount of both TPC and TFC in plant extracts is an indication of possible therapeutic role of plants and plant-based products [24,25].

The antioxidant activity is considered as one of the leading predictive indicators for potential pharmacological properties of plants. The DPPH radical scavenging is widely used assay to assess the antiradical activity of plant extracts and compounds. The antioxidant properties of plant extracts are mainly due to phenolics. The polyphenols have the ability to donate proton from hydroxyl groups to DPPH free radical to stabilize it. This reaction is accomplished by a change in colour intensity from violet to yellow as an indication of antioxidant activity [26]. The current investigation revealed a promising DPPH radical scavenging with lowest IC_50_ value for ethyl acetate fraction followed by chloroform fraction; however, a significant difference was observed in both values. The antioxidant activity of *T. dioica* extracts was most probably due to presence of polyphenolics having ability to stabilize the DPPH radical. In present study, the ethyl acetate fraction of *T. dioica* exhibited highest antioxidant activity and it was noticeable that this fraction also contained the highest phenolic and flavonoid contents. The studies have shown that DPPH radical scavenging by plant extracts is associated with the quantity and quality of polyphenols present in particular extracts [14,27].

The α-glucosidase inhibition by ethyl acetate fraction of *T. dioica* for the current investigation was most probably due to a variety of metabolites which might be present in extract. The secondary metabolites of plants contain stereo-specific structural features which can bind with the amino acid residues of α-glucosidase to modify or reduce the enzymatic activity [14,17]. The loss, reduction, or delay in enzyme activity results in comparatively lesser digestion of carbohydrates which consequently leads to low availability of glucose for intestinal absorption [28,29]. A study worked out the α-glucosidase inhibitory activities of plants of family Apocynaceae, Clusiaceae, Euphorbiaceae, and Rubiaceae and 37 plant extracts out of 45 showed better enzyme inhibition than acarbose. The IC_50_ value of acarbose was reported as 117.20 µg/mL which was much higher than value (35.10 ± 0.33) obtained in current experiment [30]. Therefore, the IC_50_ comparison in this case may be complicated to some extent. A recent report demonstrated the comparative effect of *Catharanthus roseus* and its alkaloid, vindoline against α-glucosidase inhibition. The findings of the work clearly expressed that plant extracts and vindoline showed poor enzyme inhibition in spite of the fact having good antioxidant activities [31]. Another, species of the same family, *Tamarix aphylla* from Tunisia was reported to possess antioxidant and α-glucosidase inhibitory properties which were most probably ought to presence of compounds like gallic acid, quercetion, ferulic acid, ellagic acid and caffeic acid which were identified using HPLC-DAD [32]. However, it is well reported that the plants having good to excellent antioxidant activities also exhibit reasonable α-glucosidase inhibitions depending upon the nature of active Phyto-constituents which may vary from species to specie and depend upon the extraction procedures and climatic factors [13,14,15,33].

There is very little scientifically proved information on antidiabetic role of *T. dioica*. Therefore, identification of metabolites may provide us valuable information on the biologically active ingredients responsible for observed α-glucosidase inhibition. The most potent fraction (ethyl acetate) based on the findings of in vitro antioxidant and α-glucosidase assays, was subjected to metabolite profiling using ^1^HNMR. ^1^HNMR is a versatile technique to identify the metabolites in complex mixtures or extracts obtained from plants and many previous investigations have used this technique to profile the secondary metabolites [34,35].

The compounds were identified on the basis of their chemical shift values and *J* resolved values for duplets and multiplets. The identification revealed that there were compounds of diverse chemical classes including flavonoids, organic acids and amino acids. Some high valued compounds were explored by^1^HNMR. The region between 6 and 9 ppm typically represents the aromatic region as mostly the polyphenolic compounds show peaks in this region. The region ranging from δ_H_ 1ppm to δ_H_ 4ppm is usually termed carbohydrates and organic acid region. The amino acids also show their characteristic peaks in this region [36]. 

Most of the flavonoids and related compounds like tamarixetin, apigenin, catechin, rutin, quercetin, gardenin B, kaempferol, isorhamnetin and myricetin were identified in the ethyl acetate fraction of *T. dioica*. These compounds exhibited the peaks in aromatic region of ^1^HNMR spectrum. The amino acids were also identified from their characteristic peaks in amino acid region. The leucine, lysine, glutamic acid, aspartic acid, and tyrosine were the identified amino acids in ethyl acetate fraction.

Tamarixetin is a characteristic compound of genus *Tamarix* and known to possess significant antioxidant and antibacterial properties [37]. The apigenin is also well known medicinally active compound. A study reported the antioxidant and antidiabetic activity of apigenin rich ethanolic fraction of *Cycas revoluta* [38]. Many flavonoids were also identified in the understudy fraction. The flavonoids are polyphenols with diverse medicinal properties including α-glucosidase inhibition. Similarly, kaempferol, rutin and quercetin are well reported for α-glucosidase inhibition potential [39]. A previous report highlighted the antidiabetic properties of *Conocarpu slancifolius* and metabolite profiling identified the presence of isorhamnetin in its hydroethanolic leaf extract [29]. The presence of high value compounds in *T. dioica* ethyl acetate fraction was of significant deal to consume the plant for pharmacological benefits. The structural interactions among metabolites and protein were explored using computation tool of docking analysis.

Molecular docking analysis not only provided substantial information on the structural interactions among identified metabolites and enzyme but also provided the binding energy for favourable consideration of particular compound as α-glucosidase inhibitor. The interaction plots show that acarbose exhibited conventional hydrogen bonding with active site residues of ASP68, ASP349 and GLU304 it also showed other non-bonding interactions with HIS348, PHE300 and ARG312. Myricetin which exhibited lowest binding energy was interacting through hydrogen bonding with SER156, HIS239 and GLN350, while it showed pi-alkyl interaction with ARG312 and pi-pi interaction at PHE157 which are also key residues of the active pocket of alpha-glucosidase [38]. Rutin was also identified in the ethyl acetate fraction and docking analysis revealed conventional H-bonding with HIST279, THR307 and pi-alkyl contact with TRP242 and PRO309.Tamarixetin interacted with ARG312 residue through C-H bond and pi-alkyl strapping. Tamaridone showed conventional H-bonding with THR307 due to presence of aromatic –OH group. A pi-pi mode of interaction at HIS279 and a pi-sigma at PRO309 was also observed. Catechin exhibited H-bonding with PHE310, PRO309, GLU304 and pi-alkyl interfacing at HIS279, PHE157 and ARG312. Another important compound, kaempferol showed H-bonding with ARG349 position and C-H bonding with HIS279 whereas PHE158, PHE157, ARG312 exhibited pi-alkyl interactions. Quercetin interacted with GLN350 and ASN241 through conventional H-bonding while pi-pi stacking was noticed at PHE157 and pi-alkyl at ARG312. Isorhamnetin formed H-bonding at HIS239, PRO309 and ARG312 while pi-pi stacking at PHE157.Somenon-bonding interactions by some metabolites highlighted ARG312 and SER156 as most vulnerable sites to be interacted by metabolites. The ribbon diagrams present that both ligands are embedded deep into the active pocket of the enzyme. Conclusively docking studies gave an insight about the α-glucosidase inhibition confirming the antidiabetic attributes of the plant extract by revealing the type of bonding between protein and phytochemicals.

The docking tool has been widely adopted to predict the α-glucosidase inhibition by secondary metabolites of plants by exploring the possible hydrophilic and hydrophobic interactions. The functional groups of secondary metabolites interact with the various amino acids of enzyme to modify the structure-based function. Studies have shown the efficacy of docking studies to identify the potential amino acid sites in enzyme as potential sites for drug development [40,41]. It was also validated that there might be some variation in the pharmacological properties of plant extracts depending upon the solvent system adopted for extraction [42].

The current study provided valuable information on the metabolites in *T. dioica* and their possible role to manipulate the α-glucosidase activity due to structural interactions. However, some additional aspects may be considered, including extraction optimization, antioxidant activity, novel computational study, and in vivo investigations. The use of homology α-glucosidase model for docking studies to investigate the allosteric interaction by compounds at molecular level is a common practice; however, the identification of α-glucosidase amino acid residues and backbone moieties responsible for interacting with inhibitors must be definitive. The field may be further strengthened by the molecular aspects by investigating the allosteric regulation of enzyme using crystalline structure of α-glucosidase. The crystal structure-based docking information may provide us further details to facilitate the mechanistic investigations for manipulation of protein ligand interactions. The hydrophobic active site pockets may of great deal for novel drug design. Similarly, the antioxidant activity may be best assessed by executing in actual biochemical environments such as in vivo, which can be of significant magnitude regarding stress oriented metabolic dysfunctions.

It may be ascertained that the antioxidant activity and α-glucosidase inhibitory potential of various fractions of *T. dioica* observed in current study was due to presence of a variety of active metabolites in extracts. The mechanism behind the antidiabetic activity of flavonoids seems to involve the marking of certain important functional amino acid regions of α-glucosidase to reduce the enzyme activity. The findings confirmed the ethnopharmacological use of *T. dioica* to treat diabetes and the compounds identified were mainly responsible for medicinal potential.

## 4. Materials and Methods

### 4.1. Processing of Plant Material

The *T. dioica* plant was collected from Dera Ismail Khan region of Khyber Pakhtunkhwa province of Pakistan. The plant was identified by expert taxonomist in Botany Department Govt. College University, Lahore, Pakistan. The clean aerial parts of the plant were selected and dried in the shade for about a month. The dried plant was grinded in powder in electric grinder. 

### 4.2. Preparation of Extracts

The 1600 g of the grinded material was infused five times with methanol (containing about 10% water) at room temperature; initially, 2 dm^3^ of methanol was used and the soaking continued for three weeks and then filtered first through muslin cloth and then through filter paper with manual pressing of the residue, the residue thus obtained was repeatedly soaked with fresh methanol, amount of methanol and number of days were subsequently reduced in the next soakings and continued till no more material was extracted from plant material. All the filtrates were combined, and methanol removed until it was thick, using vacuum rotary evaporator at 45 °C. The concentrated material was dissolved in 750 cm^3^ of distilled water. The aqueous extract thus obtained was extracted several times with *n*-hexane, chloroform, ethyl acetate and n-butanol in sequence. Each extract of *n*-hexane, chloroform and ethyl acetate was concentrated in vacuum rotary evaporator at 45 °C till a highly viscous material is obtained, further dried in oven at 40–45 °C for 48 h. The n-butanol extract was concentrated and dried in oven at 60 °C for two to three weeks. The dried plant material thus obtained from each solvent was dissolved in methanol and was further investigated for its biological activities. 

### 4.3. DPPH Radical Based Antioxidant Activity

The radical scavenging or antioxidant activity was done by the DPPH radical scavenging method. Different concentrations of each solvent extract of plant ranging from 50 µg/mL to 500 µg/mL were prepared in methanol, 1 mL of each was vigorously shaken with 3 mL of methanolic solution of 0.1 mM 2,2-Diphenyl-1-picrylhydrazyl (DPPH). The mixture obtained was stored in dark for an hour. After that, the absorbance of samples was noted on spectrophotometer at 517 nm against blank which acts as control containing only DPPH solution. The radical scavenging potential of samples was compared with known antioxidant Butylated hydroxy toluene (BHT) solutions having different strengths. The free radical scavenging ability was calculated using the formula [43]:

% DPPH inhibition = [Absorbance_(control)_ − Absorbance_(sample)_/Absorbance_(control)_] × 100

### 4.4. Total Phenolic Contents

The Folin–Ciocalteu’s method was used to determine total phenolic contents of samples. To 0.1 mL of each solvent extract of plant (0.5 mg/mL of methanol) added 2.8 mL 10% Na_2_CO_3_ and 0.1 mL of FC reagent after thorough mixing and keeping for 40 min. The absorbance of reaction mixture was noted at 725 nm using spectrophotometer to calculate the amount of phenolic compounds. The standard calibration curve was formed by the absorbance values of gallic acid solution of different concentrations (ranging from 400 µg/mL to 25 µg/mL) treated in the same way as sample. The total phenolics were calculated as mg gallic acid equivalent/gm dry extract [44].

### 4.5. Total Flavonoid Contents

Initially, 0.5 mg of each solvent extract of plant was dissolved in 1 mL of methanol. To the methanolic plant extracts, 0.3 mL of 5% NaNO_2_ were added and left for 5 min. After that 0.3 mL of 10% AlCl_3_ solution was added. After 10 min, 2 mL of 1 M NaOH was added to the extract mixture. The volume of the mixture was adjusted to 10 mL by adding distilled water. The absorbance was measured at 510 nm. The total flavonoid contents expressed as quercetin equivalent in mg per gram of dry extract [45].

### 4.6. The α-Glucosidase Inhibition Assay

The α-glucosidase inhibition was performed by adopting previously reported method with slight modifications [46]. Briefly,100 µg of each solvent extract of plant was dissolved in 70 µL of 50 mM Phosphate buffer of pH 6.8 having α-glucosidase (1 unit/mL) from *Saccharomyces cerevisiae*. An incubation was carried out at 37 °C for a period of 10 min. followed by addition of 10 µL of ρ-nitrophenol glucopyranoside (5 mM) as substrate. The reaction mixture was allowed to incubate for 30 min to complete the reaction. The absorbance was noted at 405 nm for the ρ-nitrophenol released from the substrate. The acarbose was used as standard enzyme inhibitor. The % inhibition was measured by following formula,

$$\%\;inhibition=\left(Absorbance\;of\;blank-\frac{Absorbance\;of\;Control}{Absorbance\;of\;Blank}\right)\times 100$$

### 4.7. Metabolite Profiling

The most potent extract was utilized for identification of secondary metabolites. The major classes of primary and secondary metabolites present in extract were confirmed by ^1^H-NMR technique. The most potent extract was dissolved in CH_3_OH-*d*_4_ and KH_2_PO_4_ buffer of pH 6.0 in D_2_O having 0.1%. Sample was vortex for 2 min and followed by ultrasonication for 30 min at 30 °C. The centrifugation was done at 13,000 rpm for a period of 10 min. Then, 600 μL of supernatant was collected for ^1^HNMR analysis on INOVA 500 MHz spectrometer (Varian Inc., Paulo Alto, CA, USA), at 499.887 MHz frequency with tetramethylsilane (TMS) as internal standard. For peak interpretation and compound identification, the obtained spectrum was bucketed with Mestrenova 11.0 software.

### 4.8. Docking Analysis

The MOE 2016.08 (molecular operating environment) software was utilized for the in-silico docking studies. Swiss Model Server was used to generate homology modelled alpha-glucosidase structure. The sequence of amino acid of alpha-glucosidase (Code No. P53341) was retrieved from UniProt and based on the highest similarity (72.4%) 3D structure of *Saccharomyces cerevisiae* iso maltase (PDB 3AJ7) was selected as template. The pdb format of produced homology structure was downloaded to be docked with identified phytochemicals using MOE software. The binding energies of protein–ligand interaction was estimated and the interaction between proteins and ligands were studied by Discovery studio visualizer software 2020 [17].

## 5. Conclusions

The findings revealed that ethyl acetate fraction of *T. dioica* was the most potent fraction regarding scavenging of free radicals and α-glucosidase inhibition representing the moderate antidiabetic potential. The ethyl acetate fraction also exhibited the higher TPC and TFC, influencing the antioxidant and enzyme inhibitory properties. The ^1^HNMR profiling of the fraction revealed the presence of some high value compounds including flavonoids having ability to inhibit dietary enzyme inhibition as a target for diabetes management. The binding energy data highlighted the possible structural interactions among identified secondary metabolites and α-glucosidase enzyme. The myricetin and rutin find the closed proximity to standard drug acarbose on the basis of binding energy. The information obtained also confirmed the ethnomedicinal use of *T. dioica* to treat diabetes mellitus. The outcomes of this study may be helpful in developing the naturopathic approach towards metabolic disorder. The identification of metabolites also added valuable information on phytochemical database of *T. dioica*. Further investigations may be carried out on more précised and targeted fractionation and isolation of compounds for in vivo studies to develop more authentic and comprehensive picture. 

## Figures and Tables

**Table 1:** Effect of solvent on TPC and TFC of extracts from *T. dioica.*

Solvent Fraction	TPC (mg GAE/g DE)	TFC (mg QE/g DE)
*n*-Hexane	164.44 ± 4.76 ^d^	78.51 ± 0.61 ^d^
Ethyl accetate	205.45 ± 1.36 ^a^	156.85 ± 1.33 ^a^
Chloroform	188.35 ± 1.63 ^b^	111.15 ± 2.29 ^b^
*n*-Butanol	173.88 ± 2.58 ^c^	83.19 ± 0.96 ^c^

The alphabets (a–d) represents level of statistical significance. Values having different alphabets were significantly different with *p* < 0.05.

**Table 2:** The compound identity in ethyl acetate fraction based on and chemical shifts (δ_H_ ppm) along with *J* resolved values.

Sr. No.	Metabolites	Chemical Shift (δ_H_ ppm) with *J* Resolved Values
1	Tamarixetin	7.8 (dd, *J* = 8.9 Hz, 1.4 Hz)
2	D-mannitol	3.68 (dd, *J* = 8.28 Hz, 5.64 Hz); 3.84 (dd, *J* = 11.34 Hz, 1.26 Hz)
3	Gardenin B	7.90 (d, *J* = 8.9 Hz)
4	Nevadensin A	7.14 (d, *J* = 2.76 Hz); 8.03 (*J* = 8.9 Hz)
5	Tamaridone	3.90 (s); 6.20 (s)
6	Tamadone	6.77 (s); 7.47 (d, *J* = 1.92 Hz)
7	Apigenin	6.30 (s); 7.73 (d, 8.8 Hz)
8	Catechin	2.82 (dd, *J* = 23.04 Hz); 5.86 (d, *J* = 11.6 Hz)
9	Myricetin	6.18 (d, *J* = 2.04 Hz); 7.36 (s)
10	Kaempferol	6.90 (m)
11	Nonanal	1.65 (m)
12	Tetradecane	0.90 (m)
13	Rutin	3.55 (dd, *J* = 11.6 Hz, 6.3 Hz); 3.64 (dd, *J* = 13.56 Hz, 2.52 Hz)
14	Quercetin	6.89 (d, *J* = 1.86 Hz); 7.66 (d, *J* = 2.5 Hz)
15	Leucine	3.73 (m)
16	Lysine	1.39 (m)
17	Glutamic acid	2.39 (m); 2.01 (m); 2.10 (m)
18	Aspartic acid	2.80 (dd, *J* = 14.88 Hz, 7.02 Hz)
19	Serine	3.83 (dd, *J* = 15.66 Hz, 7.8 Hz)
20	Tyrosine	3.01 (dd, *J* = 19.56 Hz, 7.08 Hz) 3.17 (dd, *J* = 12.54 Hz, 5.46 Hz)
21	Isorhamnetin	7.71 (d, *J* = 31.98 Hz) 9.73 (s)
22	Vanillic acid	3.90 (s), 6.94 (d, *J* = 8.6 Hz)

**Table 3:** The binding energy data and nature of bonding exhibited by identified metabolites docked in α-glucosidase.

Sr. No.	Metabolites	Binding Energy (kJ/mol)	Hydrogen Bonding	Other Interactions
1	Tamarixetin	−14.9304		HIS279, HIS239, ARG312
2	D-mannitol	−11.4684	SER156, GLY160, LYS155, ASP408	PHE158, ASN412
3	Gardenin B	−11.8613	ARG312, SER 156	HIS279, PHE311, TYR31
4	Nevadensin A	−14.0441		GLY159, PHE311, LYS155, PHE157, PHE300, ALA278, TYR313, ARG312
5	Tamaridone	−14.8680	THR307	PRO240, HIS239, HIS279, PHE157, PRO309, VAL305
6	Tamadone	−12.9285		PHE157, HIS279, LYS155, HIS239, PRO240, ARG312
7	Apigenin	−12.6378	ASN241	HIS279, ARG312
8	Catechin	−14.3305	PHE310, PRO309, GLU304	HIS279, PHE157, ARG312
9	Myricetin	−15.3993	SER156, HIS239, GLN350	ARG312, PHE157
10	Kaempferol	−14.6258	ARG439	PHE158, HIS279, PHE157
11	Nonanal	−8.0170	ARG312	PHE311, LYS155
12	Tetradecane	−8.7492		HIS279, PHE157, HIS239, LYS155, PHE311
13	Rutin	−15.3011	HIS279, THR307	PRO309, TRP242, HIS245
14	Quercetin	−14.6382	GLN350, ASN241	HIS279, HIS239, PHE157, ARG312
15	Leucine	−9.2032		LYS155, PHE157, HIS239, PRO240
16	Lysine	−10.3665	PHE157	PHE157
17	Glutamic acid	−11.2994		ASP408, PHE157
18	Aspartic acid	−9.8152	GLY160, SER235, PHE310	GLY159
19	Serine	−9.8837	SER156, PHE310, GLY159	
20	Tyrosine	−12.0217	ASN241, PHE157	HIS239, PHE158
21	Isorhamnetin	−13.8628	PRO309, HIS239, ARG312	HIS279, PHE157
22	Vanillic acid	−10.5649	ASP408	ARG312, HIS239
23	Acarbose	−16.4212	ASP68, ASP349, GLU304	HIS348, PHE300, ARG312

## Data Availability

Not applicable.
